# The utility of synovial fluid levels of ADAMTS9 and ADAMTS4 in predicting treatment responses to intraarticular steroid injections in patients with knee osteoarthritis

**DOI:** 10.3906/sag-1809-108

**Published:** 2020-08-26

**Authors:** Kenan ÖZLER

**Affiliations:** 1 Department of Orthopedics, Konya Beyşehir State Hospital, Konya Turkey

**Keywords:** ADAMTS9, ADAMTS4, synovial fluid, WOMAC score, knee osteoarthritis

## Abstract

**Background/aim:**

This study aims to identify the role of synovial fluid levels of a disintegrin and metalloproteinase with thrombospondin motifs 9 (ADAMTS9) and a disintegrin and metalloproteinase with thrombospondin motifs 4 (ADAMTS4) for the prediction of intraarticular steroid injection success in knee osteoarthritis (OA).

**Material and methods:**

A total of eighty-four advanced stage knee OA patients (42 with stage 3 OA and 42 with stage 4 OA) were enrolled in the study. Baseline and posttreatment outcomes were determined using Western Ontario and McMaster Universities Osteoarthritis Index (WOMAC). Pretreatment synovial fluid ADAMTS9 and ADAMTS4 levels were measured by enzyme linked immunosorbent assay (ELISA). ‘’Total WOMAC score regression of 18% and above’’ was taken as a minimal clinically important difference (MCID) to indicate improvement. Determining the best predictors of intraarticular steroid injection success in both groups was evaluated by multiple logistic regression analyses.

**Results:**

Synovial fluid ADAMTS9 levels were significantly lower in the stage 4 OA group when compared with the stage 3 group. The level of synovial fluid ADAMTS9 was statistically significantly lower in the WOMAC score percent change ≥18% than the WOMAC score percent change <18% group in Stage 3 OA group (P *=* 0.026). Decreasing synovial fluid ADAMTS9 levels (odds ratio (OR): 0.625, 95% confidence interval (CI): 0.437–0.893) were found to be predictive for the WOMAC score percent change ≥18 in all OA patients (P *=* 0.010). Decreasing ADAMTS9 levels in synovial fluid (OR: 0.602; 95% CI = 0.372–0.974) were predictive for MCID in stage 3 OA patients ( *P* = 0.039).

**Conclusion:**

The lower levels of ADAMTS9 in synovial fluid may be used in conjunction with high WOMAC scores in the prediction of intraarticular steroid injection success and advanced stage knee OA patients.

## 1. Introduction

Knee osteoarthritis (OA) is a destructive joint disease in which joint manifestations include progressive loss of cartilage, cartilage calcification, osteophyte formation, and subchondral bone remodelling. Osteoarthritis is graded according to radiological findings of changes in the joints. The Kellgren and Lawrence (K&L) scoring system is used to denote the radiological stage in OA. In advanced stages of OA, progressive changes occur due to cartilage degradation [1]. The extracellular matrix (ECM) of cartilage consists of type 2 collagen and aggrecan, which is a significant proteoglycan [1]. Progressive changes in cartilage in OA are dependent on active cell processes mediated by cytokines and proteases [1]. The role of synovial inflammation in the progression of OA is well known [2]. Several studies demonstrated that inflammation of the synovial membrane played a role in the progress of joint degeneration by increasing the release of proteases, such as matrix metalloproteinases and a disintegrin and metalloproteinase with thrombospondin motifs (ADAMTS), in the fibroblasts of cartilage [3,4]. Members of the ADAMTS family, especially ADAMTS4 and ADAMTS9 are already known to bind and degrade ECM components and contribute to joint destruction processes in arthritis [5]. Zhang et al. [6] showed that ADAMTS4 levels in synovial fluid were significantly lower in late-stage OA than in early-stage OA. Peng et al. [7] reported that ADAMTS4 in synovial fluid might be a potential biomarker for cartilage degeneration. Previously, the aggrecanases ADAMTS4 and ADAMTS5 were shown to contribute to cartilage destruction and to be responsible for aggrecan degradation, especially in patients with early-stage OA [8]. Gok et al. [9] suggested that the cytosine adenine (CA) repeat polymorphism of the ADAMTS9 gene could be used to determine the radiological progression of OA.

Although recent studies have already shown that ADAMTS4 and ADAMTS9 are involved in cartilage destruction in OA [10,11], the possible role of these matrix metalloproteinases in OA progression and their utility in the evaluation of treatment success remains unclear. This study aimed to determine the significance of synovial fluid ADAMTS4 and ADAMTS9 levels in OA progression, particularly in the assessment of the response to intraarticular steroid injection in advanced stage OA patients.

## 2. Materials and methods

Eighty-four advanced stage knee OA female patients (42 with stage 3 OA and 42 with stage 4 OA) with matching body mass index (BMI) were recruited consecutively from the outpatient clinic of Konya Beyşehir State Hospital in 2018. The diagnosis of knee OA was determined according to the radiographic features. The radiological stage was based on the K&L scale, which is accepted as a reference standard of the World Health Organization. It requires the presence of all five radiological criteria: osteophytes on the joint side, periarticular ossicles, joint area narrowing (JSN), small pseudocyst regions within the subchondral bone and a change in the formation of the bone ends. Inclusion criteria of OA patients: patients with radiological features (stage 3 medium multiple osteophytes, JSN, minimal sclerosis, and deformity of bone ends) and stage 4 (giant osteophytes, evident JSN, severe sclerosis, and deformity of bone ends) [12], women who have not received any intraarticular and/or systemic OA therapy before and a BMI between 30 kg/m2 and 18 kg/m2. 

Exclusion criteria: patients with other knee joint diseases (arthroscopic anterior cruciate ligament reconstruction, meniscus angioplasty or arthroscopy, non-OA group), infectious diseases, septic arthritis, obesity, neurological or neuromuscular diseases, bone tumours, osteoporosis or trauma related fractures, diabetes mellitus, Addison’s disease, immune system disorder, rheumatological diseases and previous use of systemic steroids and intraarticular hyaluronic acid injections. Also, OA women who did not comply with stage 3 and stage 4 criteria, that is, no changes in X-ray or changes in only osteophyte or JSN, were not included in the study.

All participants provided written informed consent, and the study ethics committee approval was obtained from the local Ethical Committee of the University (approval date/number: 06.02.2018/005-006). Clinical examination was performed, and anthropometric measurements, concomitant diseases (diabetes, hypertension, and cardiovascular diseases) were recorded for all participants included in the study. Knee function was assessed before and 1 month after the treatment with intraarticular steroid injection, using the Western Ontario and McMaster Universities Osteoarthritis Index (WOMAC). The WOMAC score is composed of 24 parameters that include pain (score range: 0–20), stiffness (score range: 0–8), and functional impairment (score range: 0–68) [13]. Improvement in the WOMAC score after intraarticular injection was assessed within an average of one to 3 months. ‘’Total WOMAC score regression of 18% and above’’ was taken as a minimal clinically important difference (MCID) to indicate improvement [14].

Only advanced stage OA patients who received an intraarticular injection of the steroid methylprednisolone acetate (40 mg to 1 mL) were included in the study. Patients managed with surgical treatment were excluded. Knee joint synovial fluid samples were obtained using sterile needles. The samples were stored at –80 °C until the day of analysis. 

Synovial fluid levels of ADAMTS4 and ADAMTS9 were analyzed using Eastbiopharm branded human ADAMTS4 and ADAMTS9 enzyme linked immunosorbent assay (ELISA) kit with an immunoassay device (Immulite 2000) and presented in pg/mL and ng/mL, respectively. The blood reference range of the ADAMTS9 assay kit is 5–400 ng/mL, and the blood reference range of the ADAMTS4 assay kit is 5–1000 pg/mL. The intraassay and interassay coefficients of variation of the kit were lower than 10% and 12%, respectively.

### 2.1. Statistical analysis

Data analysis was performed using SPSS for Windows, version 22 (SPSS Inc., Chicago, IL, USA). Whether the distributions of continuous variables were normal or not was determined using the Kolmogorov–Smirnov test, homogeneity of variances was evaluated using the Levene test. Continuous variables were shown as mean ± standard deviation (SD) or median (min–max), where applicable. While Student’s t-test compared the mean differences between groups, the Mann–Whitney U test was applied for comparisons of the median values. Patients with advanced OA, according to the K&L scale, were grouped into stage 3 and stage 4 OA. The optimal cutoff points of synovial fluid ADAMTS9 levels in advanced stage OA cases were evaluated by receiver operating characteristic (ROC) analyses, calculating the area under the curve (AUC) as giving the maximum sum of sensitivity and specificity for the variables test. Stage 3 and stage 4 groups were subgrouped into those with a WOMAC score percent change ≥18% and a WOMAC score percent change <18%. Synovial fluid ADAMTS9, synovial fluid ADAMTS4 levels and other variables were compared with Student’s t-test, responding and not responding to the improvement in WOMAC score in stage 3 and stage 4 groups. Also, categorical comparisons were performed using the χ2-test. Finally, multiple logistic regression analysis was performed separately in stage 3, stage 4, and all advanced stage knee OA patients to determine the effect of independent variables associated with response to intraarticular therapy. Adjusted odds ratios (ORs) and 95% confidence intervals (CI) were calculated for each variable. A P-value of less than 0.05 was considered statistically significant.

## 3. Results

A total of 84 BMI matched OA patients with advanced stage disease (stage 3, *n* = 42; stage 4, *n* = 42) were enrolled in the study. Their baseline anthropometric and biochemical characteristics are given in Table 1. The mean age of the patients with stage 3 OA was 62.38 ± 6.78 years, and patients with stage 4 OA was 67.64 ± 5.63 years. The mean age of the patients with stage 4 OA was significantly higher than the stage 3 OA group (P *<* 0.001). There were no statistically significant differences among smokers in the groups (P *=* 0.766).

**Table 1:** Baseline characteristics and laboratory parameters of stage 3 and stage 4 OA patients.

			Stage 3 OA n = 42	Stage 4 OAn = 42	P-value*
Age (years)			62.38 ± 6.78	67.64 ± 5.63	<0.001
Smoking			7(16.7%)	8 (19.0%)	0.776
Synovial fluid ADAMTS4 (pg/mL)			128.51 ± 27.79	131.84 ± 35.47	0.633
Synovial fluid ADAMTS9 (ng/mL)			5.92 ± 1.94	5.02 ± 1.35	0.017
WOMAC score		Total score (before intraarticular injection)	82.12 ± 6.18	65.21± 16.26	0.001
		Total score (after intraarticular injection)	67.26± 7.21	53.19± 12.72	0.001
WOMAC score change (median[min–max])			13[5–31]	9 [1–40]	0.099
Concomitant diseases	Hypertension and cardiovascular diseases		7(16.7%)	11 (26.2%)	0.559
	Diabetes		4 (9.5%)	4(9.5%)	
	No additional diseases		31 (73.8%)	27 (64.3%)	

*; a P-value of < 0.05 is considered statisticallysignificant. Independent sample t-test, P-value: Statistically significant difference between stage 3 and stage 4 knee osteoarthritis, ADAMTS4; A disintegrin and metalloproteinase with thrombospondin motif 4, ADAMTS9; A disintegrin and metalloproteinase with thrombospondin motif 9, WOMAC score; Western Ontario and McMaster Universities Osteoarthritis Score.

**Figure 1:**
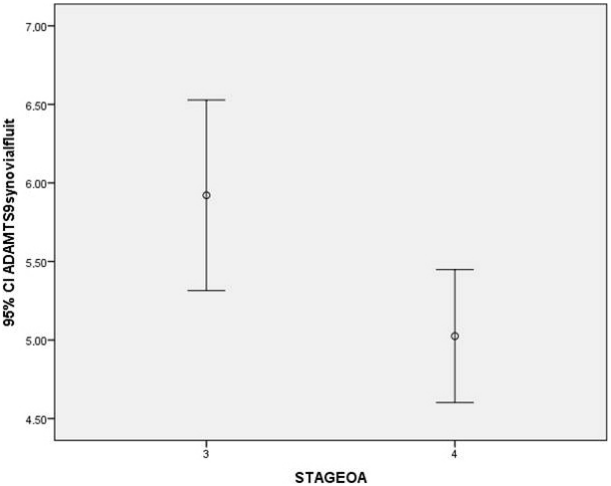
Synovial fluid ADAMTS9 levels in stage 3 and stage 4 OA patients.

Synovial fluid ADAMTS9 levels were 5.92 ± 1.94 ng/mL in stage 3 OA and 5.02 ± 1.35 ng/mL in stage 4 OA cases (Figure 1). The patients with stage 4 OA had significantly lower levels of ADAMTS9 when compared with stage 3 OA group (P *=* 0.017). WOMAC scores before intraarticular injection were 82.12 ± 6.18 and 65.21 ± 16.26. WOMAC scores after intraarticular injection were 67.26 ± 7.21 and 53.19 ± 12.72 respectively for stage 3 and stage 4 OA patients. WOMAC scores before and after intraarticular injection were significantly higher in the stage 3 OA group compared to the stage 4 OA group (P *<* 0.001and P *<* 0.001). Synovial fluid ADAMTS4 levels were 128.51 ± 27.79 pg/mL and 131,84 ± 35.47 pg/mL respectively in stage 3 and 4 OA groups. There were no statistically significant differences among synovial fluid ADAMTS4, WOMAC score change and concomitant diseases (hypertension,cardiovascular diseases, and diabetes ) between groups (Table 1).

**Figure 2:**
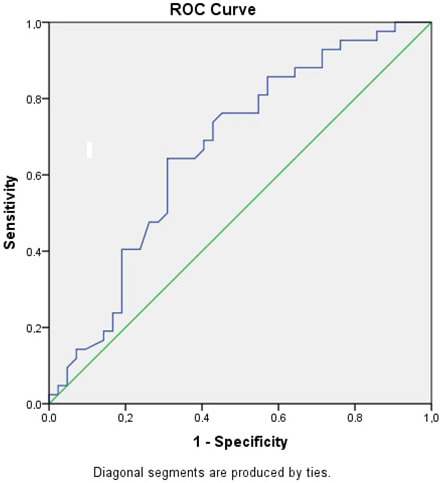
Synovial fluid ADAMTS9 ROC curve in OA patients.

Synovial fluid ADAMTS9 levels were again evaluated with ROC analysis (Figure 2); cutoff levels were determined, and AUC values were calculated. The AUC, best cutoff values, sensitivity, and specificity for distinguishing the groups for each parameter are given in Table 2. Synovial fluid ADAMTS9 levels were found to be statistically significant. When the groups that responded and did not respond to the treatment were compared within stage 3 and stage 4, the synovial fluid ADAMTS9 levels were 5.04 ± 1.44 ng/mL in the WOMAC score percent change ≥18% group, and 6.43 ± 2.01 ng/mL in the WOMAC score percent change <18% group.

**Table 2:** Best cutoff value, sensitivity, specificity, and AUC (95%Cl) of synovial fluid ADAMTS9 in OA.

	Cutoff	Spe %	Sen %	AUC (95 % CI)	P-value*
Synovial fluid ADAMTS9 (ng/mL)	5.54	71%	55%	0.666 (0.549–0.783)	0.009

AUC; Area under curve, ADAMTS9; A disintegrin and metalloproteinase with thrombospondin motif 9.

The level of synovial fluid ADAMTS9 was statistically significantly lower in the WOMAC score percent change ≥18% than the WOMAC score percent change <18% group in stage 3 OA (P *=* 0.026). There were no statistically significant differences among age, smoking, synovial fluid ADAMTS4, and concomitant diseases between the groups in stage 3 (Table 3). Age, smoking, synovial fluid ADAMTS9, synovial fluid ADAMTS4, and concomitant diseases were not statistically different between WOMAC score percent change ≥18% and WOMAC score percent change <18% in stage 4 (Table 4).

**Table 3:** Clinical and laboratory parameters according to the response of the WOMAC score percentage in stage 3 OA patients.

		WOMAC score percent change <18%	WOMAC score percent change ≥18%	P-value*
Age (years)		63.32 ± 7.32	60.50 ± 5.30	0.208
Smoking		6 (21.4%)	1(7.1%)	0.242
Synovial fluid ADAMTS4 (pg/mL)		128.79 ± 28.47	127.95 ± 27.40	0.928
Synovial fluid ADAMTS9 (ng/mL)		6.43 ± 2.01	5.04 ± 1.44	0.026
Concomitant diseases	Hypertension and cardiovascular diseases	4(14.3%)	3 (21.4%)	0.122
	Diabetes	1 (3.6%)	3 (21.4%)	
	No additional diseases	23 (82.1%)	8 (57.1%)	

*; a P-value of < 0.05 is considered statistically significant. Independent sample t-test, P-value: Statistically significant difference between WOMAC score percent change <18% and WOMAC score percent change ≥18% in stage 3 OA patients, ADAMTS4; A disintegrin and metalloproteinase with thrombospondin motif 4, ADAMTS9; A disintegrin and metalloproteinase with thrombospondin motif 9, WOMAC score; Western Ontario and McMaster Universities Osteoarthritis Score.

**Table 4:** Clinical and laboratory parameters according to the response of the WOMAC score percentage in stage 4 OA patients.

		WOMAC score percent change <18%	WOMAC score percent change ≥18%	P-value*
Age (years)		68.08 ± 6.04	67.00 ± 5.06	0.548
Smoking		6 (24.0%)	2 (11.8%)	0.282
Synovial fluid ADAMTS4 (pg/mL)		127.47 ± 28.99	138.27 ± 43.47	0.339
Synovial fluid ADAMTS9 (ng/mL)		5.22 ± 1.42	4.60 ± 1.11	0.139
Concomitant diseases	Hypertension and cardiovascular diseases	6 (24%)	5 (29.4%)	0.774
	Diabetes	3 (12%)	1(5.9%)	
	No additional diseases	16 (64%)	11(64.7)	

*; a P-value of <0.05 is considered statistically significant. Independent sample t-test, P-value: Statistically significant difference between WOMAC score percent change <18% and WOMAC score percent change ≥18% in stage 4 OA patients, ADAMTS4; A disintegrin and metalloproteinase with thrombospondin motif 4, ADAMTS9; A disintegrin and metalloproteinase with thrombospondin motif 9, WOMAC score; Western Ontario and McMaster Universities Osteoarthritis Score.

### 3.1. Multivariate analysis

At first, logistic regression analysis was performed to determine the variables associated with an 18% improvement in the WOMAC (MCID) of intraarticular steroid injection in all OA patients. Decreasing synovial fluid ADAMTS9 levels (OR: 0.625, 95% CI: 0.437–0.893) were found to be predictive for the MCID in all OA patients (P *=* 0.010) (Table 5).

**Table 5:** Regression analysis for the prediction of poor treatment success in all advanced stage OA patients.

	WOMAC score percent change (≥18%)	
	OR (95 % CI)	P-value*
Age (years)	0.967 (0.905–1.034)	0.324
Smoking	2.732 (0.706–10.572)	0.146
Synovial fluid ADAMTS4 (pg/mL)	1.005 (0.991–1.020)	0.447
Synovial fluid ADAMTS9 (ng/mL)	0.625 (0.437–0.893)	0.010
Concomitant diseases	0.568 (0.221–1.464)	0.242

*; a P-value of <0.05 is considered statisticallysignificant.ADAMTS4; a disintegrinandmetalloproteinasewiththrombospondin motif 4, ADAMTS9;A disintegrin and metalloproteinase with thrombospondin motif 9, WOMAC score; Western Ontario and McMaster Universities Osteoarthrtis Index.

Also, logistic regression analysis was performed again to determine the variables associated with an 18% improvement in the WOMAC (MCID) of intraarticular steroid injection treatment in stage 3 and stage 4 OA patients, respectively. Only decreasing ADAMTS9 levels in synovial fluid (OR: 0.602; 95% CI = 0.372–0.974) were predictive for MCID in stage 3 OA patients (P = 0.039) (Table 6). Age, smoking, synovial fluid ADAMTS9, synovial fluid ADAMTS4, and concomitant diseases were not statistically associated with MCID in stage 4 (Table 6). 

**Table 6:** Regression analysis for the prediction of poor treatment success in stage 3 and stage 4 OA patients, respectively.

	WOMAC score percent change (≥16%)			
	Stage 3		Stage 4	
	OR (95 % Cl)	P-value*	OR (95% CI)	P-value*
Age (years)	0.937 (0.848–1.036)	0.206	0.966 (0.864–1.079)	0.538
Smoking	3.545 (0.383–12.817)	0.265	2.368 (0.417–13.461)	0.331
Synovial fluid ADAMTS4 (pg/mL)	0.999 (0.976–1.023)	0.926	1.009 (0.991–1.027)	0.332
Synovial fluid ADAMTS9 (ng/mL)	0.602 (0.372–0.974)	0.039	0.651 (0.365–1.163)	0.147
Concomitant diseases	0.290 (0.069–1.216)	0.090	1.031 (0.285–3.735)	0.963

*; a P-value of <0.05 is considered statistically significant. ADAMTS4; A disintegrin and metalloproteinase with thrombospondin motif 4, ADAMTS9;A disintegrin and metalloproteinase with thrombospondin motif 9, WOMAC score; Western Ontario and McMaster Universities Osteoarthrtis Score.

## 4. Discussion 

Advanced stage OA is a chronic disease associated with progressive cartilage degeneration. Known triggers of OA include mechanical factors, age, and inflammation [15]. In recent years, neither systemic nor intraarticular medical treatment is effective in stopping the progression of OA [15]. Surgical treatment is the last resort in patients with OA. 

Several studies have focused on factors in the progression of OA in the elderly population. Kevorkian et al. [16] showed that the expression of ADAMTS1, ADAMTS5, ADAMTS9, and ADAMTS15 genes was significantly decreased and that the expression of ADAMTS16, ADAMTS2, ADAMTS14, and ADAMTS12 genes was increased in OA cartilage, compared to normal cartilage. Naito et al. [17] demonstrated that ADAMTS4 might play an important role in the degradation of aggrecan in human osteoarthritic cartilage. In our study, we found no significant differences in synovial fluid ADAMTS4 levels of stage 3 and stage 4 OA patients. However, the ADAMTS9 levels in the synovial fluid of stage 4 OA patients were significantly lower than those of stage 3 OA patients. Also, the WOMAC score in stage 4 OA group was significantly lower than in the stage 3 OA group. Pretreatment WOMAC scores were higher in the stage 3 OA group when compared with the stage 4 OA group, possibly pointing out the ongoing cartilage degeneration.

In human chondrocyte cell lines, Coughlan et al. [10] showed that both ADAMTS5 and ADAMTS9 appeared to be important in cartilage ECM formation and turnover, and that depletion of ADAMTS5 and ADAMTS9 will lead to improved ECM formation and neocorticalization. Yaykasli et al. [11] demonstrated that leptin increased the expression of ADAMTS4, ADAMTS5, and ADAMTS9 genes and cartilage degeneration in human chondrocytes. Yang et al. [18] revealed abnormal increases in Cysteine-rich protein 61(Cyr61) and ADAMTS4 protein levels in OA tissues and chondrocytes. In the same study, Cyr61 reduced ADAMTS4 protein expression in chondrocytes, and Cyr61 interacted with ADAMTS4 to affect OA progression. Stanton et al. [19] found no significant phenotypic abnormalities in ADAMTS4 and ADAMTS5 knockout mice. Still, they found a significant reduction in the severity of surgically induced OA in ADAMTS5 knockout groups compared with ADAMTS4 knockout groups [19]. The present study demonstrates the prediction of intraarticular treatment success in stage 3 OA patients with synovial fluid ADAMTS9 levels. Intraarticular steroid injection was ineffective in stage 4 OA patients due to the severity of cartilage degeneration.

In conclusion, we think that ADAMTS9’s low level of synovial fluid is significant in the stage 3 group where cartilage degeneration is continuing, and is meaningless in the stage 4 group where cartilage has wholly degenerated, there is no longer any extracellular matrix structure and there will be no regeneration. With this result, the significantly low level of ADAMTS9 may be related to the fact that intraarticular or systemic treatments will still be effective due to the ECM protease in the synovial fluid of stage 3 OA patients. 

The limitations of our study are mainly the lack of evaluation of other proteases and proteoglycans, which are risk factors of OA, a small number of patients and the controls had a history of joint injury and had been operated upon the knee for different reasons. The lack of a control group and early-stage OA is the limitation of the study. WOMAC 20,50, and 70 indexes could not be obtained due to the limited number of patients. The fact that no questionnaire related to the psychosocial status of patients has been conducted is another limitation of our study. Further prospective studies in larger cohorts are needed to validate the results of the present study. 

## Acknowledgments

The authors would like to thank the staff at Beyşehir State Hospital and to all patients who participated in the study.
